# Foods, Nutrients and Dietary Patterns in Relation to Irrational Beliefs and Related Psychological Disorders: The ATTICA Epidemiological Study

**DOI:** 10.3390/nu13051472

**Published:** 2021-04-27

**Authors:** Christina Vassou, Mary Yannakoulia, Ekavi N. Georgousopoulou, Christos Pitsavos, Mark Cropley, Demosthenes B. Panagiotakos

**Affiliations:** 1 Department of Nutrition and Dietetics, School of Health Sciences and Education, Harokopio University, 176 71 Athens, Greece; cvassou@hua.gr (C.V.); myianna@hua.gr (M.Y.); 2 School of Medicine Sydney, University of Notre Dame, Sydney, NSW 2007, Australia; ekavigeorgousopoulou@gmail.com; 3 First Cardiology Clinic, School of Medicine, University of Athens, 115 27 Athens, Greece; chrpits@outlook.com; 4 School of Psychology, University of Surrey, Guildford GU2 7XH, UK; mark.cropley@surrey.ac.uk

**Keywords:** irrational beliefs, anxiety, depression, dietary habits, dietary patterns, the Mediterranean diet, the Western-type dietary pattern

## Abstract

We explored the differences in dietary habits and dietary patterns between individuals characterized by irrational beliefs with no or low anxiety and depressive symptoms and individuals characterized by irrational beliefs with high anxiety and depressive symptomatology. Within the context of the ATTICA cohort study (2002–2012), 853 participants without evidence of cardiovascular disease (453 men (45 ± 13 years) and 400 women (44 ± 18 years)) underwent mental health assessment through the irrational beliefs inventory (IBI), the Zung self-rating depression scale (ZDRS) and the state–trait anxiety inventory (STAI). Demographic characteristics, a thorough medical history, dietary behaviour and other lifestyle behaviours were also evaluated and analysed using factor analysis. Five main factors related to dietary patterns were extracted for the high-IBI/low-STAI group of participants (explaining the 63% of the total variation in consumption), whereas four factors were extracted for the high-IBI/high-STAI participants, the high-IBI/low-ZDRS participants and the high-IBI/high-ZDRS participants, explaining 53%, 54% and 54% of the total variation, respectively. A Western-type dietary pattern was the most dominant factor for individuals reporting irrational beliefs and anxiety or depressive symptomatology. The high refined carbohydrates and fats dietary pattern was the most dominant factor for individuals with irrational beliefs but without psychopathology. Linear regression analysis showed that irrational beliefs, in combination with anxiety or depression, age, sex and BMI, were important predictors of adherence to the Mediterranean diet. Dietary habits interact with irrational beliefs and, in association with the consequent psychological disorders, are associated with overall diet, and presumably may affect the health status of individuals.

## 1. Introduction

In recent years, nutrition research has shifted its focus from foods and nutrients to dietary patterns and whole-of-diet analysis, a holistic approach that better reflects people’s habits. Foods are consumed in complex combinations based on dietary patterns, and their balance in consumption plays a significant role due to the interactions across various nutrients and bioactive constituents [1]. In the majority of studies, the prevailing dietary patterns identified are the healthy (prudent) dietary pattern and the unhealthy dietary pattern. A healthy and balanced dietary pattern, in terms of reduced mortality and morbidity and improved well-being, is high in the consumption of unprocessed vegetables, fruits, whole grain products, low-or non-fat dairy products, seafood, legumes and nuts, moderate in alcohol drinking, low in red and processed meat, and low in sugar-sweetened foods, beverages and refined grains [2]. The Mediterranean diet, along with the dietary approaches to stop hypertension (DASH) diet and the vegetarian (or semi-vegetarian) diet are considered to be among the healthiest diets recommended by several health organizations [2]. Unhealthy dietary patterns, such as the Western-type dietary pattern (WDP), are characterized by the excessive consumption of red and processed meat, carbonated foods and beverages, fried foods, high-fat dairy products, eggs, refined cereals, potatoes and corn, and a low consumption of fruits, vegetables, whole grains, fish, nuts and seeds [3]. However, many people today tend to adopt a mixed or balanced dietary pattern, including both plant and animal foods. Moreover, several other dietary hypotheses have been investigated so far in relation to quality of life and mental health, as well as the prevention of diet-related diseases, including: low-carbohydrate versus high-fat dietary patterns, high-carbohydrate versus low-fat dietary patterns, high-protein versus low-fat dietary patterns, low glycaemic diets, etc. [4,5,6]. 

The relationship between mental health and dietary habits is bidirectional; mood affects eating habits, and eating habits affect mood and psychological well-being [7]. Several epidemiological studies have indicated the significant role of diet in mental health, acting either as positive and protective health behaviour or as a negative and risky behaviour that has been associated with several complications [8,9,10,11]. Particularly in relation to irrational beliefs, which arise from a fundamental process of absolutist thinking and pessimism [12], they are considered to be the leading cause of psychopathological conditions (e.g., anxiety and depression), and subsequently may contribute to dysfunctional behavioural responses, such as the adoption of an unhealthy diet, overeating, substance overuse, eating disorders, etc. [13]. Ellis placed irrational beliefs at the core of rational emotive behaviour therapy (REBT), arguing that these are the primary reasons for human suffering and dysfunction [12]. We can change our emotional response to events from unhealthy to positive, which better aids us in achieving life goals by helping us in confronting, questioning and acting against our irrational beliefs. However, the association between irrational beliefs and dietary habits is not well understood and appreciated. 

Therefore, the current study sought to explore the associations between individuals’ eating habits (in terms of macronutrients, foods and dietary patterns) and irrational beliefs, as well as related psychological emotions, anxiety or depression. To our knowledge, this is the first study that attempts to expand knowledge on how specific psychological statuses based on dysfunctional beliefs express different dietary behaviours. 

## 2. Materials and Methods

### 2.1. Design

The ATTICA study is a population-based, prospective survey (2002–2012) that was carried out in the province of Attica, in Greece. In total, 3042 adults (18–89 years old, 49% men (73% participation rate)) with no clinical evidence of cardiovascular diseases, other atherosclerotic diseases or chronic viral infections agreed to give blood samples for biochemical and genetic analyses, in addition to their socio-demographic, lifestyle and medical details, including a psychological assessment. 

### 2.2. Setting

The study was conducted in the greater metropolitan Athens area (including 78% urban regions and 22% rural regions) during the years ranging from 2001–2002. Each participant was interviewed/examined face-to-face, in their home or place of work, by qualified professionals (cardiologists, general practitioners, dieticians and nurses, as well as psychiatrists/psychologists).

### 2.3. Sample

Of the initially enrolled 3042 participants, a sub-sample of 853 participants (453 men (45 ± 13 years) and 400 women (44 ± 18 years)) agreed to participate in the psychological evaluation within this study. This sub-sample is representative of the total study sample, and there were no differences in sex and age distribution between the studied sample and the overall study population (all *p*-values > 0.4). 

### 2.4. Bioethics

The Institutional Ethics (#017/1.5.2001) committee approved the study and all participants were informed about the aims and procedures and agreed to participate, providing written consent. 

### 2.5. Baseline Measurements

#### 2.5.1. Socio-Demographic and Lifestyle

The socio-demographic and lifestyle characteristics examined included age, sex, educational attainment and mean annual income during the past three years, dietary habits, physical activity status and smoking habits. Socio-economic status (SES) was also classified into three groups (tertiles; low, medium and high) according to the SES categorization already used, taking into account education level and the mean annual income over the preceding 3 years [14]. 

#### 2.5.2. The Food Frequency Questionnaire 

Dietary habits were assessed using a validated, semi-quantitative food frequency questionnaire (FFQ), the EPIC–Greek questionnaire provided by the Unit of Nutrition of Athens University Medical School, and according to which participants recalled and reported average weekly or daily intakes of food items over the past year [15,16]. The questionnaire assessed the usual dietary intake of 156 foods and beverages typically consumed in Greece, with seven non-overlapping response categories. Pictures assisted the responders in defining the portion sizes in many foods that were included in the questionnaire. Non-refined cereals and products (such as whole-grain bread, pasta, rice, etc.), vegetables, legumes, fruit, dairy products (such as cheese, yogurt, milk), nuts, potatoes, eggs, sweets, fish, poultry, red meat and meat products and use of olive oil in cooking, as well as coffee, tea and alcohol intake, were all calculated on a weekly basis over the past year. The frequency of consumption was quantified in terms of the number of times a month a food was consumed in small, medium or large portion sizes. Food intakes were extrapolated into macronutrient intakes by using food composition tables. Energy intake was calculated by summing the energy intake from macronutrients and alcohol, assuming 4 kcal/g for carbohydrates and proteins, 9 kcal/g for lipids and 7 kcal/g for alcohol. 

#### 2.5.3. Adherence to the Mediterranean Diet, MedDietScore

A diet score (MedDietScore) was calculated based on the reported food intake and assessed adherence to the Mediterranean diet for each participant [16]. In particular, for the consumption of items presumed to be “close” to this pattern (i.e., those suggested daily or for more than four servings per week), we assigned a score of zero when someone reported no consumption, a score of 1 when they reported a consumption of 1 to 4 times per month, 2 for 5 to 8 times, 3 for 9 to 12, 4 for 13 to 18 and a score of 5 for more than 18 times per month. For the consumption of food items presumed to be “away” from this diet (such as meat and meat products) we assigned the scores on a reverse scale (i.e., 0 when a participant reported almost daily consumption and 5 for rare or no consumption). Especially for alcohol, we assigned a score of 5 for the consumption of fewer than 3 wine glasses per day, a score of zero for the consumption of more than 7 wineglasses per day and scores of 1 to 4 for the consumption of 3, 4–5, 6 and 7 wine glasses per day, respectively. Higher values (range 0–55) of this special diet score indicate a greater adherence to the Mediterranean diet [16]. 

#### 2.5.4. Assessment of Physical Activity Using the International Physical Activity Questionnaire (IPAQ)

The short form of the International Physical Activity Questionnaire (IPAQ) was used to assess physical activity status. IPAQ was used as an index of weekly energy expenditure using frequency (times per week), duration (in minutes per time) and intensity of sports or other habits related to physical activity (in expended calories per time) [17]. Sedentary participants were defined as those who did not report any physical activities, while the rest of the participants were classified as physically active. 

#### 2.5.5. Smoking Habits Evaluation

Current smokers were classified as individuals who smoked at least one cigarette per day during the previous year, former smokers were defined as those who had quit smoking more than 1 year previously, and the rest were classified as never smokers.

### 2.6. Clinical and Biochemical Evaluation

Participants with fasting blood glucose >125 mg/dL during the examination or who reported taking anti-diabetic medication were defined as having diabetes, according to the American Diabetes Association guidelines for the diagnosis of type-2 diabetes mellitus [18]. Blood glucose levels (mg/dL) were measured with a Beckman Glucose Analyser (Beckman Instruments, Fullerton, CA, USA). Obesity was defined as a body mas index (BMI) greater than 29.9 Kg/m^2^ [19]. Arterial blood pressure was measured three times, with the participant in a sitting position and being at rest for at least 30 min. Participants who had an average blood pressure of ≥140/90 mmHg or were under anti-hypertensive medication were classified as being hypertensive. Hypercholesterolemia was defined as having total cholesterol levels >200 mg/dL or the use of lipids-lowering agents [20].

### 2.7. Psychological Evaluation

Irrational beliefs were assessed at baseline using the Irrational Beliefs Inventory (IBI), a brief self-report measure based on the work of Ellis [21]. The inventory consists of 11 statements, each reflecting one irrational belief, including, worrying, rigidity, need for approval, problem avoidance and emotional irresponsibility [22]. Each item is followed by a 9-point bipolar scale ranging from disagree to agree. The scales are summed to yield a total score ranging from 0 to 88 (the higher the score, the greater the severity of irrational beliefs). The IBI was developed as an instrument to assess the association between the endorsement of irrational beliefs and various aspects of maladaptive emotion and behaviour that have been developed within Ellis’s theoretical and applied model, which views irrational beliefs as maladaptive [21]. 

The validated Greek translation of the Zung Self-Rating Depression Scale (ZDRS) was used to measure depressive symptomatology. The time window was the preceding 4-week period before the administration. The ZDRS total score range is 20–80, with higher values indicating more severe depression symptoms [23]. We applied a cut-off score of 45 to dichotomize the study cohort into participants with and without clinically relevant depressive symptomatology, based on the validated ZDRS cut-off score for the Greek population [24]. The validated Greek translation of the state anxiety sub-scale of the Spielberger State–Trait Anxiety Inventory (STAI) was used to assess anxiety levels [25]. The total score of the 20-item STAI ranges from 20 to 80, with higher score values being indicative of more severe anxiety symptoms [23]. In the context of this study, the STAI score was used as a continuous variable, since cut-off scores for the adult Greek population require further validation [25]. 

For this study, the high-IBI/low-STAI–high-IBI/high-STAI variable was created, classifying participants with a high IBI score (i.e., above the median value, 52, which is 50% of the participants) and a high STAI score (>40, median) into one group and those with a high IBI score but a low STAI score into another. Similarly, the high-IBI/low-ZDRS–high-IBI/high-ZDRS variable was created classifying participants with a high IBI and a high ZDRS (>34, median) into one group and those with a high IBI and a low ZDRS into the other. It was observed that the 66% of participants who had a high IBI score (i.e., above the median value of 52) also had a high STAI score (>40, median) (68% men vs. 64% women, *p* = 0.08). Similarly, the 65% of individuals with a high IBI score also had a high ZDRS score (>34, median) (68% men vs. 62% women, *p* = 0.01).

### 2.8. Study Size, Power Analysis

Power analysis showed that the number of participants in the working dataset was adequate to evaluate two-sided differences between the sub-groups of the study and the investigated parameters greater than 20%, achieving statistical power >0.80 at <0.05 probability level (*p*-value).

### 2.9. Statistical Methods

#### Descriptive Statistics and Hypothesis Testing

Continuous variables are presented using mean values and ± standard deviation (SD) (or median (1st, 3rd quartile) if normality was not met), while categorical variables use frequencies. Associations between categorical variables were tested using the chi-squared test. For group comparisons between normally distributed continuous variables, the Student’s t-test was used, while for those that were not normally distributed the Mann–Whitney non-parametric test was applied. P-P plots were used to test for normality in continuous variables. 

Multiple linear regression analysis evaluated the association between adherence to the Mediterranean diet and the irrational beliefs, depression and anxiety of the participants, taking into consideration various socio-demographic, lifestyle and clinical characteristics. Results are presented as b-coefficients and 95% confidence intervals. The variance inflation factor (VIF) was used to evaluate potential co-linearity between the independent variables (variables with VIF > were excluded). The Durbin–Watson criterion was applied to test for the serial dependency of the independent variables, and the scatter plot of residuals versus the fitted values was used to evaluate homoscedasticity.

### 2.10. Multivariate, Pattern Analysis

Factor analysis, using the principal component method, was applied to identify dietary patterns among the four psychological categories (high-IBI/low-STAI, high-IBI/high-STAI, high-IBI/low-ZDRS and high-IBI/high-ZDRS). From the entire food database, 27 food categories were selected to represent all major food groups. The correlation matrix (instead of the co-variance) was preferred in order to account for the variety in the food measurement scale. The Kaiser–Meyer–Olkin test (a measure of sampling adequacy for performing factor analysis) was relatively high (i.e., 0.61), indicating a relatively good inter-relationship between the food variables, suggesting that the data are suitable for factor analysis [26]. Food groups entered in the analysis were coded as servings per month. All Eigenvalues derived from the correlation matrix of the standardized variables were considered (an eigenvalue indicates the amount of the variance in consumption explained by each factor). A seventeen-factor solution was extracted, but the first five were considered and interpreted here, as they explained most of the variance in consumption. These factors (patterns) were interpreted (named) according to the scores of each food group that correlated with it most (i.e., scores > 0.4) (factor scores are interpreted similarly to correlation coefficients (i.e., higher absolute values indicate that the food variable contributes most to the construction of the factor). 

STATA software, version 16 (TStat S.r.l. 67039 Sulmona AQ, Italy) was used for all statistical analyses.

## 3. Results

### 3.1. Baseline Characteristics in Relation to Irrational Beliefs, Anxiety and Depression

The mean IBI score was 53 ± 11 for men and 53 ± 10 for women (*p* = 0.83), the mean STAI score was 40 ± 11 for men and 42 ± 12 for women (*p* = 0.05) and the mean ZDRS score was 43 ± 10 for men and 47 ± 9 for women (*p* < 0.001). Participants with a high IBI score (i.e., above the median value, 52 (i.e., 50% of the participants)) and a high STAI score (>40, median) were classified in one group (high-IBI/high-STAI) and those with a high IBI score but a low STAI score were classified into another (high-IBI/low-STAI). Similarly, participants with a high IBI and a high ZDRS (>34, median) were classified into one group (high-IBI/high-ZDRS) and those with a high IBI and a low ZDRS were classified into the other (high-IBI/low-ZDRS). 

It was observed that the 66% of participants who had a high IBI score (i.e., above the median value, 52) also had a high STAI score (>40, median) (68% men vs. 64% women, *p* = 0.08). Similarly, 65% of individuals with a high IBI score also had a high ZDRS score (>34, median) (68% men vs. 62% women, *p* = 0.01). Moreover, the participants with irrational beliefs combined with anxiety or depression were mostly female, older, less educated, more physically active, obese and smokers compared to participants with irrational beliefs but without severe anxiety and depressive symptomatology (all *p*-values < 0.05) (Table 1).

### 3.2. Energy, Macronutrients and Food Intake According to Irrational Beliefs, Anxiety and Depression

In Table 2, energy, macronutrient intake and the consumption of specific food groups are presented via irrational beliefs and depression or anxiety status. Participants with high irrational beliefs and anxiety or depression symptoms consumed more potatoes and red meat compared to those participants with high irrational beliefs but low or no anxiety or depression symptoms (all *p* values < 0.05). On the contrary, participants with high irrational beliefs and low anxiety or depression consumed more low-fat dairy products than did individuals with high irrational beliefs and anxiety or depression (all *p* values = 0.001). Moreover, participants with high irrational beliefs but low or no anxiety consumed olive oil more often than those with high irrational beliefs and anxiety, whereas participants with high irrational beliefs but low depression consumed olive oil less often than those with high irrational beliefs and depression (all *p* values < 0.001). 

To conclude, individuals with high irrational beliefs and anxiety or depressive symptomatology consumed more carbohydrates per day relative to those reporting low levels of anxiety or depression and consumed more saturated fats than those with low or no depression symptoms (all *p* values < 0.05). 

### 3.3. Dietary Patterns According to Irrational Beliefs, Anxiety and Depression

As reported above, the main goal of this analysis was to extract dietary patterns according to irrational beliefs status. Factor analysis identified 17 factors (dietary patterns) in total; however, five principal factors were identified for the participants with high-IBI/low-STAI scores that explained 63% of the total variation in consumption and four factors for high-IBI/high-STAI (that explained 53% of the total variation), high-IBI/low-ZDRS (that explained 54% of the total variation) and high-IBI/high-ZDRS (that explained 54% of the total variation), respectively. Table 3 and Table 4 show the results for each factor (scores with an absolute value greater than 0.50 are presented in bold; the higher the absolute value, the higher the participation of a specific food or food group in the development of the factor).

The following factors, which are characterized by the main consumption of the food groups, have been derived:

High-IBI/low-STAI.

Factor 1: High refined carbohydrates and fats pattern.

Factor 2: High-protein and fats pattern.

Factor 3: Meat and light soft drinks consumption pattern.

Factor 4: Alcohol drinking pattern, low in sugar-sweetened soft drinks.

Factor 5: Pattern based on dairy products.

Factor 1 was the most prevailing dietary pattern and explained 19% of the total variation. Each of the remaining four factors explained from 8% (factor 5) to 14% (factor 2) of the variation in intake.

High-IBI/high-STAI.

Factor 1: Western-type dietary pattern.

Factor 2: High in light soft drinks and fats pattern.

Factor 3: Fruits and fish pattern.

Factor 4: Low-fat dairy products pattern.

Factor 1 was the most dominant dietary pattern and explained 20% of the total variation. Each of the remaining three factors explained from 9% (factor 4) to 12% (factor 2) of the variation in intake.

High-IBI/low-ZDRS.

Factor 1: High refined carbohydrates and fats pattern.

Factor 2: High-carbohydrate, nuts and alcohol fats pattern. 

Factor 3: Meat-based pattern.

Factor 4: Pattern based on dairy products and sugar-sweetened carbonated drinks. 

Factor 1 was the most dominant dietary pattern and explained 19% of the total variation. Each of the remaining three factors explained from 9% (factor 4) to 14% (factor 2) of the variation in intake.

High-IBI/high-ZDRS.

Factor 1: Western-type dietary pattern. 

Factor 2: High in light soft drinks and fats pattern. 

Factor 3: “Healthy” dietary pattern. 

Factor 4: Low-fat dairy pattern, low in alcohol. 

Factor 1 was the most prevailing dietary pattern and explained 21% of the total variation. Each of the remaining three factors explained from 9% (factor 4) to 12% (factor 2) of the variation in intake.

Thus, emotional disorders (in addition to irrational beliefs) predicted a diet close to a “Western” dietary pattern (i.e., high consumption of red meat, high-fat dairy products, butter, sweets, refined grains), whereas, the absence or lowered levels of anxiety and depression (in addition to irrational beliefs) predicted a less unhealthy dietary profile. 

### 3.4. Adherence to the Mediterranean Diet in Relation to Irrational Beliefs, Anxiety and Depression Status 

An age-adjusted, inverse correlation was observed between IBI score and MedDietScore (r = −0.11, *p* = 0.01). Similarly, MedDietScore was inversely correlated with STAI (r men = −0.11, *p* < 0.001, r women = −0.20, *p* < 0.001), but was positively correlated with ZDRS (r men = 0.13, *p* < 0.001, r women = 0.17, *p* < 0.001). Regarding the association between adherence to the Mediterranean dietary pattern and irrational beliefs, in conjunction with anxiety and depression, it was observed that individuals with a high IBI and a low STAI scored 28 ± 6 in the MedDietScore index, whereas individuals with a high IBI and a high STAI scored much lower (23 ± 7 (*p* < 0.001)). However, individuals with a high IBI and a low ZDRS scored 23 ± 5 in the MedDietScore index, while those with a high IBI but a high ZDRS scored higher (i.e., 26 ± 7 (*p* < 0.001)). 

Multiple linear regression analysis was used to examine the association between adherence to the Mediterranean diet and the IBI-STAI or ZDRS status of the participants, taking into consideration age, sex, socio-economic status, smoking habits and physical activity, as well as any history of hypertension, hypercholesterolemia, diabetes and obesity in order to account for potential confounding. The analysis revealed that participants with a high IBI had a lower MedDietScore as compared to those with a low IBI (b coefficient = −0.95, 95% CI −1.24, −0.66, *p* = 0.001). Moreover, participants with a high IBI but low STAI scores had a higher MedDietScore as compared to those with high-IBI/high-STAI scores (b coefficient = 1.15, 95% CI 0.6, 1.7, *p* = 0.001). However, participants with a high IBI but low ZDRS scores had a lower MedDietScore as compared to those with high-IBI/high-ZDRS scores (b coefficient = −2.72, 95% CI −3.48, −1.95, *p* = 0.001). 

Based on the aforementioned conclusions, adherence to the Mediterranean diet was low among those reporting high irrational beliefs compared to those reporting low irrational beliefs. It was further lowered among those who had also anxiety symptoms in addition to irrational beliefs, but it was higher among those with depression symptoms and irrational beliefs, as compared to those without depression.

## 4. Discussion

An analysis of macronutrient, food and pattern dietary intake, under the prism of irrational beliefs and emotional disorders, was applied in a group of adults with no known diagnoses. It was revealed that dietary habits correlate with irrational beliefs and, in association with the consequent psychological symptoms, they presumably affect the health status of individuals. In particular, it was clearly observed that people reporting irrational beliefs have unhealthier dietary habits, as compared to those without significant irrational beliefs and disorders. It was also revealed that individuals with irrational beliefs who succumb to anxiety were more prone to adopt unhealthier eating habits, whereas individuals with irrational beliefs and depressive symptomatology were more likely to adopt healthier eating habits, as compared to those without depression but with irrational beliefs. 

The present analysis is unique, as it attempted to test eating habits in relation to the co-existence of irrational beliefs with emotional disorders in a group of individuals from the general population; this approach has several risks and causality cannot be determined. Based on the applied dietary patterns analysis, it was revealed that the “Western” pattern diet was the most dominant for individuals with irrational beliefs and anxiety or depressive symptomatology. On the contrary, the high refined carbohydrates and fats dietary pattern (including nuts, dry fruits, sweets, soft and cola drinks, full-fat dairy products, seed oil and margarine) was the most dominant pattern for individuals with irrational beliefs but without severe emotional problems. Interestingly, when the analysis was based on an a-priori dietary pattern using the MedDietScore (instead of the a-posterior factor analysis), it was found that, although adherence to the Mediterranean diet was low among those with high irrational beliefs, the presence of depression symptoms seems to “prevent” individuals with irrational beliefs from adapting an unhealthy diet. The latter did not happen among those with anxiety symptoms in addition to irrational beliefs. Nevertheless, the findings demonstrate a general unhealthy diet in individuals who present with psychological distress symptoms. 

Despite the limited literature associating dietary patterns with psychological disorders [27,28], some studies have indicated that following the Mediterranean and lacto–vegetarian diet, as well as eating vegetables, fruits and nuts and avoiding unhealthy snacks and meat, are linked to a lower risk of depression and other mental health conditions [27,29]. However, the “Western” dietary pattern raises the risk for mental health problems such as depression [30]. In addition, nutrients (such as ω-3PUFA) are essential for brain function, since they regulate chemical processes (e.g., metabolic pathways, neurotransmitter synthesis and cell–cell signalling) [31,32]. Our findings are consistent with the aforementioned literature; individuals with high irrational beliefs and anxiety or depression consume more red meat compared to individuals with irrational beliefs who do not experience psychopathological conditions, whereas the dietary patterns they mostly follow do not include seafood. 

There are multitudinous biological pathways that explain the connection between diet and mental health, including inflammation, oxidative stress and hippocampus [33]. A fatty-food diet increases the risk for depression and anxiety [34,35]. In particular, a Western dietary pattern can result in elevated levels of circulating plasma Aβ that may trigger hippocampal dysfunction, while a diet high in sugar and fat (or just sugar) may damage hippocampal function as well [34]. On the contrary, the healthy pattern may improve cognitive function and reduce oxidative stress [36]. 

As described, most studies to date focus on how eating habits, nutrients and dietary patterns/behaviours influence mental health, rather than the reverse. However, individuals’ dietary preferences and behaviours are associated with personal beliefs and expectations, in addition to food availability, repetitive behaviours and learning mechanisms [37]. It is considered that irrational beliefs inevitably contribute to negative psychological outcomes (e.g., anxiety and depression) and, in turn, to irrational coping mechanisms (e.g., unhealthy nutrition, a sedentary lifestyle, etc.) and adverse health outcomes [11]. In particular, negative beliefs (e.g., low self-efficacy, the perception of one’s inability to control personal life and health and the perception of many life obstacles and side effects) have been found to consistently undermine the adoption of healthy eating habits [38]. Negative emotions (like anger, loneliness, anxiety and depression) have been identified as significant antecedents and triggers of eating problems [39]. Anxiety, in particular, increases appetite and leads to a desire for foods high in fat and sugar, or overeating as a coping mechanism to distract from distressing and stressful situations [40]. Furthermore, depression has been associated with a lack of motivation to engage in healthy behaviours [40]. Additionally, individuals with anxiety or depression are characterized by carbohydrate craving, an excessive degree of carbohydrate consumption (not in vegetables, fruits, legumes, whole grains and other fibres), due to an increase in brain serotonin synthesis, even though carbohydrates are the main source of energy in the brain [41]. This finding is supported by our study; people with high irrational beliefs and anxiety or depressive symptomatology consume more carbohydrates per day than those with low levels of anxiety or depression. It is important to note that our findings show that in individuals with irrational beliefs without psychopathology, a high-carb dietary behaviour is dominant as well. 

The blocking model, according to which problematic eating behaviours are used to block out short-term awareness of negative emotions that individuals find difficult to tolerate, may explain the tendency of people with strong irrational beliefs and psychopathology to engage in unhealthy eating behaviours [39]. However, the relief obtained through unhealthy eating behaviours is only temporary, resulting not merely in acute distress [39], but also in medical conditions such as obesity and other chronic diseases (type 2 diabetes, cardiovascular diseases, etc.). 

Finally, as compared to individuals with irrational beliefs but without depressive symptomatology, the presence of depression symptoms—but not anxiety—seems to be associated with adherence to a Mediterranean dietary pattern. Depressive disorder is usually more severe and recognizable than anxiety [42]; therefore, we hypothesize that individuals with depressive symptomatology may promptly seek information or consult a health professional. Since the benefits of a healthy diet against depression have been recognized for decades, these individuals likely have adopted healthy dietary habits as a result of a medical recommendation.

### Limitations

Because of the cross-sectional design employed in this study, we cannot extract a clear direction in the research hypothesis (i.e., whether dietary habits are determinants of psychological disorders or the opposite). Therefore, in the present study we avoided such an analysis (by the exception of the regression models that evaluated adherence to the Mediterranean dietary pattern in relation to irrational beliefs and emotional disorders). In addition, residual confounding attributable to unmeasured factors always exists in epidemiological analyses. Moreover, the study sample is likely to be a limitation of the current work, although no differences in age, sex distribution and SES level were found between those who participated in the psychological evaluation and the rest of the ATTICA study participants. The baseline nutritional assessment was only performed once and it may be subject to measurement error or affected by seasonal variation and reproducibility of the collected information. However, the FFQ used was found to be reproducible and reliable, while the sampling took over a year, therefore including, on average, food choices during all seasons. Finally, we did not evaluate the presence of an eating disorder that could be a possible confounding variable, since patients with eating disorders justify refusing food by arguing nutritional concerns. 

## 5. Conclusions

We consider that our study provides important information in the field of nutrition psychology, since it maps the eating behaviours of groups with specific psychological traits that have not been studied before, and shows that even when psychopathological characteristics are absent, irrational beliefs (i.e., precursors of anxiety disorders and depression) continue to be accompanied by unhealthy eating behaviours.

## Figures and Tables

**Table 1:** Socio-demographic and clinical characteristics of participants with a high IBI score (≥52), who also had anxiety symptoms (i.e., STAI > 40) or depression (ZDRS > 34) (*n* = 853).

	Irrational Beliefs and Anxiety			Irrational Beliefs and Depression		
	Participants with High-IBI/Low-STAI	Participants with High-IBI/High-STAI	*p*	Participants with High-IBI/Low-ZDRS	Participants with High-IBI/High-ZDRS	*p*
Age, years	40 ± 10	45 ± 13	<0.001	42 ± 11	44 ± 13	<0.001
Male sex, %	52.2	47.4	0.08	53.4	46.8	0.01
Years of school	16 ± 4	10 ± 2.7	<0.001	15 ± 1	10 ± 3	<0.001
Current smokers, %	35.8	43.3	0.006	35	43.8	0.001
Physically active, %	46.8	39.6	0.009	48	39	0.001
MedDietScore (0–55)	28 ± 6	23 ± 7	<0.001	27 ± 6	23 ± 7	<0.001
Obesity, %	49.2	65.8	<0.001	51.9	64.4	<0.001
Hypertension, %	24.4	32.7	0.002	26	32	0.02
Hypercholesterolemia, %	35.8	40.3	0.10	36	40	0.11
Diabetes, %	2.5	9	<0.001	8.8	6.6	0.14

**Table 2:** Food groups and nutrient consumption according to participants with a high IBI score (≥52), who also had anxiety symptoms (i.e., STAI > 40) or depression (ZDRS > 34) (*n* = 853).

	Irrational Beliefs & Anxiety			Irrational Beliefs & Depression		
	Participants with High-IBI/Low-STAI	Participants with High-IBI/High-STAI	*p*	Participants with High-IBI/Low-ZDRS	Participants with High-IBI/High-ZDRS	*p*
Vegetables, servings/week	36.2 ± 14	35 ± 14	0.31	37 ± 14.6	34.4 ± 13.4	0.09
Legumes, servings/week	5 ± 3.2	5.1 ± 2.6	0.71	5.1 ± 3.3	5.1 ± 2.5	0.79
Non-refined cereals and products, servings/week	52.2 ± 16	54 ± 18	0.32	52.4 ± 16	53.9 ± 18	0.44
Potatoes, servings/week	10.2 ± 5.2	12.5 ± 7.4	0.002	10.4 ± 5.6	12.3 ± 7	0.02
Fruits, servings/week	26.4 ± 13	28 ± 14	0.09	27 ± 13.2	27.8 ± 13.7	0.22
Dry fruits, servings/week	6.7 ± 6	6.7 ± 6.4	0.66	6.7 ± 5.8	6.8 ± 6.4	0.63
Meat, servings/week	5.4 ± 2.2	5.8 ± 3	0.42	5.4 ± 2.2	5.8 ± 3	0.29
Red meat, servings/week	4.1 ± 4	4.6 ± 2.5	0.03	4.1 ± 1.9	4.6 ± 2.5	0.03
Poultry, servings/week	1.2 ± 0.8	1.2 ± 0.8	0.25	1.3 ± 0.8	1.2 ± 0.8	0.23
Fish and fisheries, servings/week	2.1 ± 1	2.1 ± 1	0.11	2.1 ± 0.9	2 ± 1	0.03
Eggs, servings/week	1.1 ± 0.9	1.1 ± 1.1	0.60	1 ± 1	1 ± 1	0.39
Nuts, servings/week	1.6 ± 1.6	1.6 ± 1.6	0.68	1.6 ± 1.5	1.6 ± 1.6	0.56
Sweets, servings/week	5.0 ± 2.3	4.9 ± 2.4	0.45	4.9 ± 2.3	5 ± 2.4	0.84
Dairy products, servings/week	11.6 ± 4.4	12 ± 5.3	0.81	11.5 ± 4.4	12 ± 5.3	0.62
Low-fat dairy, servings/week	3.6 ± 3.5	2.6 ± 3.2	0.001	3.6 ± 3.5	2.6 ± 3.2	0.001
**Fats and Oils**						
Olive oil (daily use, %yes)	92	81.3	<0.001	36.6	63.4	<0.001
Seed oil (daily use, %yes)	15	16	0.69	32.6	67.4	0.74
Butter (daily use, %yes)	75	76	0.64	34.6	76	0.99
Margarine (daily use, %yes)	54	56.6	0.50	31	69	0.28
**Soft drinks**						
Soft drinks, glasses/day	2.3 ± 2.4	2.6 ± 2.8	0.47	2.3 ± 2.4	2.7 ± 2.8	0.21
Cola drinks, glasses/day	1.5 ± 1.7	1.7 ± 1.8	0.46	1.5 ± 1.7	1.7 ± 1.8	0.18
**Other drinks**						
Coffee, cups/day	1.2 ± 0.6	1.3 ± 0.5	0.09	1.2 ± 0.6	1.3 ± 0.5	0.60
Alcohol, g/day	9.8 ± 14.3	8.1 ± 13.6	0.16	10 ± 14.6	8 ± 13.4	0.89
Beer, glass/day	2.1 ± 0.8	1.8 ± 0.8	0.001	2 ± 0.8	1.8 ± 0.8	0.008
Wine, glass/day	2.3 ± 0.8	2 ± 0.8	<0.001	2.3 ± 0.7	2 ± 0.8	<0.001
**Energy & macronutrient intake**						
Energy (kcal/day)	2147 ± 674	2279 ± 911	0.07	2141 ± 685	2278 ± 898	0.10
Carbohydrates (g/day)	193 ± 66	211 ± 88	0.04	194 ± 67	210 ± 86	0.04
Total proteins (g/day)	77 ± 25	81 ± 33	0.24	76.3 ± 25	81 ± 33	0.10
Total lipids (g/day)	84.5 ± 31	89.4 ± 38.6	0.17	84 ± 31	89.6 ± 38	0.08
Monounsaturated fatty acids (g/day)	57.3 ± 22	60 ± 26.4	0.16	57.3 ± 22.4	60 ± 26	0.13
Polyunsaturated fatty acids (g/day)	16.4 ± 7.3	17 ± 9.7	0.94	16.4 ± 7.5	17 ± 9.5	0.73
Saturated fatty acids (g/day)	33.3 ± 14	35.4 ± 17	0.10	32.6 ± 13.5	35.8 ± 17	0.02

**Table 3:** Factor coefficients loadings regarding foods or food groups consumed by Greek ATTICA study participants with a high IBI score (≥52), who also had anxiety symptoms (i.e., STAI > 40) (*n* = 853) at baseline.

	Participants with High-IBI/Low-STAI ^a^					Participants with High-IBI/High-STAI ^b^			
Food/Food Group	1	2	3	4	5	1	2	3	4
Vegetables	−0.493	0.459	0.192	0.134	0.144	0.374	0.346	0.435	−0.158
Fruits	−0.185	0.257	0.373	−0.056	0.059	0.337	0.246	**0.487**	0.127
Dry fruits	**0.643**	0.432	−0.127	0.075	0.227	0.467	0.177	0.093	**−0.633**
Sweets	**0.657**	0.127	−0.022	−0.238	0.129	**0.490**	0.299	−0.150	0.154
Soft drinks, other than cola drinks	**0.682**	0.139	0.014	**−0.639**	0.035	**0.674**	−0.008	**−0.562**	−0.156
Soft drinks light	−0.163	0.268	**0.661**	−0.007	−0.023	0.085	**0.593**	0.141	−0.154
Cola drinks	**0.603**	0.078	0.028	**−0.636**	0.040	**0.683**	0.000	−0.439	−0.231
Cereals	0.105	0.463	0.056	0.090	0.255	0.391	0.235	0.406	0.012
Legumes	−0.197	0.285	−0.386	0.354	0.227	0.398	0.261	0.416	−0.283
Potatoes	0.407	0.475	0.309	0.050	−0.104	**0.556**	0.226	−0.334	0.224
Poultry	−0.219	0.414	**0.644**	0.074	−0.089	**0.487**	0.080	−0.344	0.302
Red meat	0.236	**0.497**	**0.509**	0.151	−0.445	**0.728**	0.136	−0.136	0.256
Eggs	0.241	0.060	0.275	0.145	−0.260	0.105	0.262	0.147	0.279
Full-fat dairy products	**0.565**	0.226	−0.239	−0.024	0.070	0.379	0.243	0.090	0.038
Low-fat dairy products	−0.440	−0.010	0.340	−0.065	**0.711**	−0.040	0.286	0.277	**0.625**
Fish and fisheries	−0.108	0.201	−0.167	0.266	−0.451	0.226	0.099	**0.495**	−0.118
Olive oil	**−0.603**	**0.608**	−0.292	−0.313	−0.120	**0.528**	**−0.743**	0.287	0.144
Seed oil	**0.558**	**−0.605**	0.372	0.348	0.138	**−0.528**	**0.743**	−0.287	−0.144
Nuts	**0.548**	0.400	−0.181	0.144	0.229	**0.530**	0.135	0.121	−0.624
Butter	**−0.560**	**0.600**	−0.376	−0.350	−0.058	**0.562**	**−0.711**	0.318	0.159
Margarine	**0.594**	−0.519	0.354	0.288	0.033	−0.432	**0.702**	−0.227	−0.071
Ethanol intake (g/day)	0.380	0.444	−0.354	0.441	0.004	0.254	0.182	0.197	−0.182
Wine	0.167	0.300	−0.279	**0.661**	0.241	0.228	0.089	0.399	−0.150
Beer	0.348	0.355	−0.294	0.387	0.204	0.154	0.087	0.145	−0.242

Score coefficients are similar to the correlation coefficient, with higher absolute values indicative of a higher correlation between the (food) variable and the respective factor. High factor scores are presented in bold. ^a^ A high refined carbohydrates and fats pattern (Factor 1), high-protein and fats pattern (Factor 2), meat and light soft drinks consumption pattern (Factor 3), alcohol drinking pattern low in sweetened, carbonated drinks (Factor 4) and pattern based on dairy products (Factor 5). ^b^ A Western-type dietary pattern (Factor 1), high in light soft drinks and fats pattern (Factor 2), fruits and fish pattern (Factor 3) and low-fat dairy products pattern (Factor 4).

**Table 4:** Factor coefficients loadings regarding foods or food groups consumed by Greek ATTICA study participants with a high IBI score (≥52), who also had depression symptoms (ZDRS > 34) (*n* = 853) at baseline.

	Participants with High-IBI/Low-ZDRS ^a^				Participants with High-IBI/High-ZDRS ^b^			
Food/Food Group	1	2	3	4	1	2	3	4
Vegetables	**−0.520**	0.400	0.228	0.009	0.298	0.163	**0.600**	−0.142
Fruits	−0.175	0.200	0.357	0.121	0.286	0.060	**0.579**	0.067
Dry fruits	**0.514**	**0.570**	−0.129	0.076	**0.487**	0.222	0.074	0.314
Sweets	**0.589**	0.180	−0.029	0.302	**0.490**	0.367	−0.098	−0.020
Soft drinks, other than cola drinks	**0.633**	0.070	0.035	**0.495**	**0.709**	0.158	−0.459	0.318
Soft drinks light	−0.185	0.202	0.667	0.032	0.013	**0.548**	0.324	0.153
Cola drinks	**0.543**	0.007	0.042	0.502	0.713	0.147	−0.333	0.341
Cereals	−0.025	0.462	0.081	0.177	0.392	0.159	0.398	−0.307
Legumes	−0.152	0.421	−0.348	−0.054	0.310	0.164	**0.542**	0.110
Potatoes	0.296	**0.499**	0.353	−0.122	**0.569**	0.307	−0.251	−0.153
Poultry	−0.318	0.266	**0.669**	−0.100	**0.512**	0.138	−0.200	−0.309
Red meat	0.085	0.396	**0.580**	−0.441	**0.744**	0.190	−0.063	−0.175
Eggs	0.230	−0.015	0.428	−0.247	0.136	0.211	0.179	−0.417
Full-fat dairy products	**0.504**	0.274	−0.168	0.063	0.402	0.248	0.130	0.105
Low-fat dairy products	−0.406	0.079	0.353	**0.587**	−0.215	0.172	0.385	**0.560**
Fish and fisheries	0.000	0.184	−0.083	**−0.600**	0.152	−0.117	**0.588**	0.115
Olive oil	−0.732	0.429	−0.274	0.107	**0.554**	**−0.770**	0.133	0.036
Seed oil	**0.685**	−0.439	0.357	−0.115	−0.554	**0.770**	−0.133	−0.036
Nuts	0.436	**0.542**	−0.184	0.040	**0.537**	0.176	0.108	0.268
Butter	**−0.686**	0.443	−0.358	0.180	**0.579**	−0.751	0.176	0.038
Margarine	**0.709**	−0.368	0.344	−0.153	−0.459	**0.723**	−0.081	−0.232
Ethanol intake	0.350	**0.601**	−0.300	−0.268	0.244	0.161	0.217	**−0.491**
Wine	0.229	**0.477**	−0.242	−0.199	0.213	−0.023	0.414	**−0.534**
Beer	0.318	**0.571**	−0.253	−0.077	0.115	0.130	0.120	−0.342

Score coefficients are similar to the correlation coefficient, with higher absolute values indicative of a higher correlation between the (food) variable and the respective factor. High factor scores are presented in bold. ^a^ A high refined carbohydrates and fats pattern (Factor 1), high-carbohydrate, nuts and alcohol fats pattern (Factor 2), meat-based pattern (Factor 3) and pattern based on dairy products and sugar-sweetened, carbonated drinks (Factor 4). ^b^ A Western-type dietary pattern (Factor 1), high in light soft drinks and fats pattern (Factor 2), healthy dietary pattern (Factor 3) and low-fat dairy pattern low in alcohol (Factor 4).

## Data Availability

The data that support the findings of this study are available from the ATTICA study but restrictions apply to the availability of these data, which were used under license for the current study, and so are not yet publicly available. Data are however available from the authors upon reasonable request and with permission of the ATTICA project.
